# Editorial: Precision medicine in viral hepatitis: progress and prospects towards elimination

**DOI:** 10.3389/fcimb.2024.1378423

**Published:** 2024-02-19

**Authors:** Amanda Fernández-Rodríguez, Pamela Valva

**Affiliations:** ^1^ Unit of Viral Infection and Immunity, National Center for Microbiology (CNM), Health Institute Carlos III (ISCIII), Madrid, Spain; ^2^ Centro de Investigación Biomédica en Red en Enfermedades Infecciosas (CIBERINFEC), Instituto de Salud Carlos III (ISCIII), Madrid, Spain; ^3^ Multidisciplinary Institute for Investigation in Pediatric Pathologies (IMIPP), CONICET-GCBA, Laboratory of Molecular Biology, Pathology Division, Ricardo Gutiérrez Children’s Hospital, Buenos Aires, Argentina

**Keywords:** viral hepatitis, biomarkers, treatment, microbiota, disease progression, immune response

Viral hepatitis poses a significant global public health challenge, impacting millions of individuals through both acute and chronic infections, that can lead to conditions such as cirrhosis and liver cancer. Despite notable progress in medical advancements, certain aspects of its pathogenesis, immune response, and treatment remain areas of contention. The analysis of host-pathogen interactions provides a pathway to identify biomarkers crucial for diagnosis, prognosis, and understanding of disease progression. To maximize these advance’s success, it is necessary to develop and use new molecular, bioinformatic, statistical, and technical approaches to implement these tools for precision medicine.

This Research Topic aims to bring greater visibility to key issues addressed in both basic and clinical research, exploring essential topics that tackle current challenges and pave the way for future research directions. The ultimate goal is to expedite viral elimination through a comprehensive understanding of the disease and the strategic application of innovative tools and approaches.

This compilation encompasses eight articles that underscore the dynamic strides being taken in comprehending and tackling the challenges associated with hepatitis in general, and mostly in hepatitis B virus (HBV). These articles delve into treatment modalities, immune response mechanisms, biomarker identification, and the influence of microbiota changes on both the immune system and disease progression. Together, they provide significant insights into diverse facets of hepatitis, offering a comprehensive overview of the current landscape in the field.

This Research Topic sheds light on new approaches to identify metabolic risk in patients infected with hepatic viruses. Zhao et al. explored the intricate interplay between metabolism and viral hepatitis, introducing two risk assessment models grounded in metabolic pathways as valuable tools to assess disease progression and prognosis. Authors used publicly available mRNA expression profiles from nearly 500 patients to perform single-sample gene set enrichment analysis to screen and select the metabolic pathways related to risk and prognosis. By incorporating the estimated infiltration of immune cells and single-cell transcriptomics data, authors suggested that the dysfunction of liver metabolism cause immune dysregulation and highlighted the crucial role of metabolic dysfunction of NK, macrophage, and CD8+ T subsets in disease development. Thus, this study adds a nuanced layer of understanding to the complex metabolic landscape associated with viral hepatitis.

The progression of viral hepatitis has been widely studied, but their correlation with gut microbiota has not been fully clarified. Therefore, Yang et al.’s performed a gut microbiome data meta-analysis from nearly 1000 patients infected by HBV, HCV and HEV and corroborates the significant decrease of gut microbial diversity during the infection and hepatitis progression. Thus, they identified potential microbial markers, such as *Butyricimonas, Escherichia-Shigella, Lactobacillus, and Veillonella*, to predict the risk of viral hepatitis.

Currently, the risk of progression of liver disease to hepatocarcinoma (HCC) is higher in HBV infected patients, since it is one of the most important contributors to the HCC pathogenesis. Gut microbiome and their metabolic-related products, such as butyrates, play pivotal role in HCC progression, as they promote cell metabolism and may enhance the immunosuppressive tumor environment, among others. Therefore, Yan et al. deepen the correlation between gut microbiota and the immunosuppressive response to predict occurrence and prognosis of HBV-HCC. The study identifies microbial markers and also unveils the intricate interplay between gut microbiota and T-cell immune responses.

These studies open the door to new therapeutic approaches for chronic HBV patients, considering that microbiota dysbiosis can be addresed by different approaches. Probiotics, live microorganisms with healthy effects, contribute to microbiota regulation and appear to exhibit antiviral activity by preventing chronic HBV progression. Thus, Shi et al. delve into the intriguing relationship between probiotic therapy and the risk of HCC in patients with hepatitis B-related cirrhosis undergoing antiviral treatment with entecavir or tenofovir. Their findings indicate a potential protective role of probiotics, paving the way for future clinical research avenues.

Nowadays, nucleos(t)ide analogues are highly efficient at suppressing viral replication, but unfortunately HBV is never fully eliminated, and therefore new curative strategies have to be investigated to achieve HBV functional cure. However, there is a lack of the perfect *in vivo* models to correctly address the research of antivirals and immunomodulatory agents. Thus, Roca Suarez et al. performed an interspecies transcriptomic analysis that sheds light on early responses to HBV exposure in both humans and macaques. Through the identification of shared and divergent host responses, this research lays the groundwork for understanding immune variations crucial for developing effective antiviral strategies. Meanwhile, Wen et al. delve into the pursuit of a clinical cure for chronic hepatitis B, making a specific comparison between outcomes in chronic inactive carriers and patients with prior experience with nucleoside analogs. The study highlights the effectiveness of Peg-IFNα-2b in both groups, underscoring its potential, particularly for individuals with a baseline HBsAg below 100 IU/ml.

Despite treatment, reactivation can occur spontaneously or by antiviral treatment discontinuation, leading to acute-on-chronic liver failure (ACLF) which is a complex syndrome that results in a short-term mortality rate up to 90%. ACLF can develop at any stage of cirrhosis, whose treatment is based on an artificial liver support system (ALSS) which requires several procedures and high costs. Therefore, Liu et al. addressed the challenges associated to predict HBV-related ACLF. Through the analysis of clinical parameters and laboratory biomarkers, the study establishes a multi-subgroup predictive model that offers accurate prognostic information, aiding in postoperative risk stratification. This model allows to assess hospital outcomes before and after each operation session of ALSS treatment, guiding clinicians to choose the best treatment plan.

Yu et al. conducted a long-term follow-up study of patients with chronic hepatitis B. They developed a model that incorporates serum AFP and aminotransferases, demonstrating its effectiveness in assessing disease progression. This model serves as a valuable tool for outpatient follow-up, offering clinicians a practical means of monitoring and managing the ongoing health of these patients.

Overall, this compilation reflects the collaborative efforts of researchers around the world, expanding the frontiers of hepatitis research. As we celebrate these advances, we look forward to continuing to work toward the ultimate goal of eliminating viral hepatitis.

## Author contributions

AFR: Conceptualization, Project administration, Writing – original draft, Writing – review & editing. PV: Conceptualization, Project administration, Writing – original draft, Writing – review & editing.

